# (*E*)-1-(2,4-Dichloro­phen­yl)-3-[3-(4-meth­oxy­phen­yl)-1-phenyl-1*H*-pyrazol-4-yl]prop-2-en-1-one

**DOI:** 10.1107/S1600536811044400

**Published:** 2011-10-29

**Authors:** Hoong-Kun Fun, Ching Kheng Quah, Shridhar Malladi, Raghavendra Hebbar, Arun M. Isloor

**Affiliations:** a X-ray Crystallography Unit, School of Physics, Universiti Sains Malaysia, 11800 USM, Penang, Malaysia; b Medicinal Chemistry Division, Department of Chemistry, National Institute of Technology-Karnataka, Surathkal, Mangalore 575 025, India

## Abstract

In the title mol­ecule, C_25_H_18_Cl_2_N_2_O_2_, the dihedral angles between the pyrazole ring and its N- and C-bonded benzene rings are 8.28 (11) and 40.89 (10)°, respectively. The dihedral angle between the benzene rings is 39.03 (11)°. The title mol­ecule exists in a *trans* conformation with respect to the acyclic C=C bond. In the crystal, mol­ecules are linked into inversion dimers by pairs of inter­molecular C—H⋯O hydrogen bonds, generating *R*_2_^2^(14) loops.

## Related literature

For related structures and background references to pyrazole derivatives, see: Fun *et al.* (2011*a* ▶,*b* ▶). For hydrogen-bond motifs, see: Bernstein *et al.* (1995 ▶). For standard bond-length data, see: Allen *et al.* (1987 ▶).
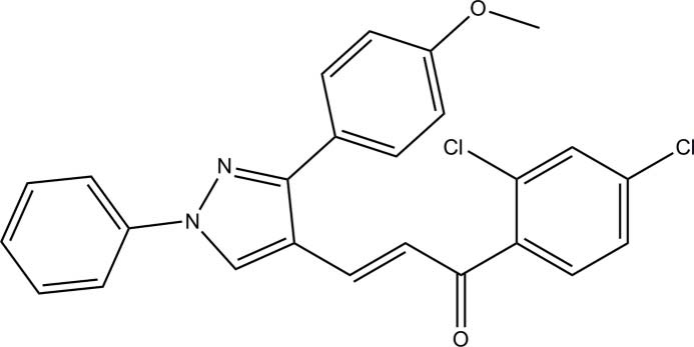


## Experimental

### 

#### Crystal data

C_25_H_18_Cl_2_N_2_O_2_
*M* *_r_* = 449.31
Monoclinic, 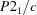

*a* = 11.5037 (9) Å
*b* = 9.9197 (8) Å
*c* = 19.6867 (16) Å
β = 94.986 (2)°
*V* = 2238.0 (3) Å^3^
*Z* = 4
Mo *K*α radiation
μ = 0.31 mm^−1^
*T* = 296 K
0.42 × 0.26 × 0.20 mm

#### Data collection

Bruker SMART APEXII DUO CCD diffractometer
Absorption correction: multi-scan (*SADABS*; Bruker, 2009 ▶) *T*_min_ = 0.879, *T*_max_ = 0.940
24117 measured reflections
6481 independent reflections
3796 reflections with *I* > 2σ(*I*)
*R*_int_ = 0.031

#### Refinement

*R*[*F* ^2^ > 2σ(*F* ^2^)] = 0.053
*wR*(*F* ^2^) = 0.155
*S* = 1.02
6481 reflections
281 parameters
H-atom parameters constrained
Δρ_max_ = 0.30 e Å^−3^
Δρ_min_ = −0.40 e Å^−3^

### 

Data collection: *APEX2* (Bruker, 2009 ▶); cell refinement: *SAINT* (Bruker, 2009 ▶); data reduction: *SAINT*; program(s) used to solve structure: *SHELXTL* (Sheldrick, 2008 ▶); program(s) used to refine structure: *SHELXTL*; molecular graphics: *SHELXTL*; software used to prepare material for publication: *SHELXTL* and *PLATON* (Spek, 2009 ▶).

## Supplementary Material

Crystal structure: contains datablock(s) global, I. DOI: 10.1107/S1600536811044400/hb6463sup1.cif

Structure factors: contains datablock(s) I. DOI: 10.1107/S1600536811044400/hb6463Isup2.hkl

Supplementary material file. DOI: 10.1107/S1600536811044400/hb6463Isup3.cml

Additional supplementary materials:  crystallographic information; 3D view; checkCIF report

## Figures and Tables

**Table 1:** Hydrogen-bond geometry (Å, °)

*D*—H⋯*A*	*D*—H	H⋯*A*	*D*⋯*A*	*D*—H⋯*A*
C11—H11*A*⋯O1^i^	0.93	2.35	3.271 (2)	171

Symmetry code: (i) 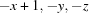.

**Fig. 1.**
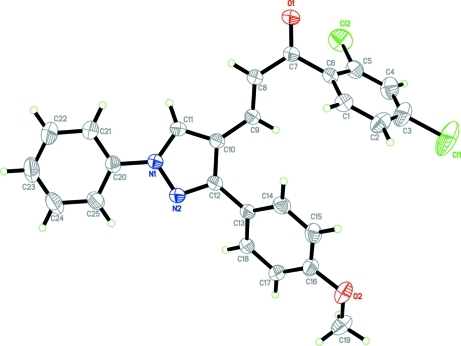
The molecular structure of the title compound showing 30% probability displacement ellipsoids for non-H atoms.

**Fig. 2.**
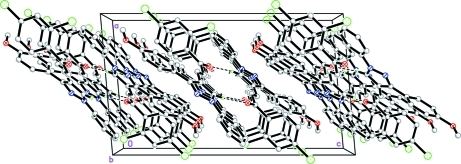
The crystal structure of the title compound, viewed along the b axis. H atoms not involved in hydrogen bonds (dashed lines) have been omitted for clarity.

## supplementary crystallographic information

### Comment

As part of our ongoing studies of pyrazole derivatives (Fun *et al.*, 2011*a*,*b*), we have synthesized the title compound to study its crystal structure.

In the title molecule (Fig. 1), the benzene (C20-C25) ring and the two phenyl (C1-C6 and C13-C18) rings form dihedral angles of 8.28 (11), 52.12 (11) and 40.89 (10)°, respectively, with the pyrazole ring (N1/N2/C10-C12). The benzene ring also forms dihedral angles of 56.27 (12) and 39.03 (11)° with dichloro-bound phenyl (C1-C6) and methoxy-bound phenyl (C13-C18) rings, respectively. The phenyl rings form a dihedral angle of 87.40 (11)°. The title molecule exists in *trans* configuration with respect to the acyclic C8═C9 bond [bond length = 1.331 (2) Å]. Bond lengths (Allen *et al.*, 1987) and angles are within normal ranges and are comparable to related structures (Fun *et al.*, 2011*a*,b).

In the crystal (Fig. 2), molecules are linked into inversion dimers by pairs of intermolecular C11–H11A···O1 hydrogen bonds (Table 1), generating fourteen-membered D^2^_2_(14) ring motifs (Bernstein *et al.*, 1995).

### Experimental

To a cold, stirred mixture of methanol (20 ml) and sodium hydroxide (12.09 mmol), 2,4-dichloroacetophenone (4.03 mmol) was added. The reaction mixture was stirred for 10 min. 3-(4-Methoxyphenyl)-1-phenyl-1H-pyrazole- 4-carbaldehyde (4.03 mmol) was added to this solution followed by tetrahydrofuran (30 ml). The solution was further stirred for 2 h at 293 K and then at room temperature for 5 h. It was then poured into ice cold water. The resulting solution was neutralized with dil. HCl. The solid that separated out was filtered, washed with water, dried and crystallized from ethanol to yield colourless blocks. Yield: 1.45 g, 80.55 %. *M.p.* : 447-449 K.

### Refinement

All H atoms were positioned geometrically and refined using a riding model with C–H = 0.93 or 0.96 Å and *U*_iso_(H) = 1.2 or 1.5 *U*_eq_(C). A rotating-group model was applied for the methyl group.

### Crystal data

**Table:**

C_25_H_18_Cl_2_N_2_O_2_	*F*(000) = 928
*M**_r_* = 449.31	*D*_x_ = 1.334 Mg m^−^^3^
Monoclinic, *P*2_1_/*c*	Mo *K*α radiation, λ = 0.71073 Å
Hall symbol: -P 2ybc	Cell parameters from 4457 reflections
*a* = 11.5037 (9) Å	θ = 2.6–24.4°
*b* = 9.9197 (8) Å	µ = 0.31 mm^−^^1^
*c* = 19.6867 (16) Å	*T* = 296 K
β = 94.986 (2)°	Block, colourless
*V* = 2238.0 (3) Å^3^	0.42 × 0.26 × 0.20 mm
*Z* = 4	

### Data collection

**Table:**

Bruker SMART APEXII DUO CCD diffractometer	6481 independent reflections
Radiation source: fine-focus sealed tube	3796 reflections with *I* > 2σ(*I*)
graphite	*R*_int_ = 0.031
φ and ω scans	θ_max_ = 30.0°, θ_min_ = 1.8°
Absorption correction: multi-scan (*SADABS*; Bruker, 2009)	*h* = −16→16
*T*_min_ = 0.879, *T*_max_ = 0.940	*k* = −11→13
24117 measured reflections	*l* = −27→27

### Refinement

**Table:**

Refinement on *F*^2^	Primary atom site location: structure-invariant direct methods
Least-squares matrix: full	Secondary atom site location: difference Fourier map
*R*[*F*^2^ > 2σ(*F*^2^)] = 0.053	Hydrogen site location: inferred from neighbouring sites
*wR*(*F*^2^) = 0.155	H-atom parameters constrained
*S* = 1.02	*w* = 1/[σ^2^(*F*_o_^2^) + (0.059*P*)^2^ + 0.6136*P*] where *P* = (*F*_o_^2^ + 2*F*_c_^2^)/3
6481 reflections	(Δ/σ)_max_ = 0.001
281 parameters	Δρ_max_ = 0.30 e Å^−^^3^
0 restraints	Δρ_min_ = −0.40 e Å^−^^3^

### Special details

**Table:**

Geometry. All esds (except the esd in the dihedral angle between two l.s. planes) are estimated using the full covariance matrix. The cell esds are taken into account individually in the estimation of esds in distances, angles and torsion angles; correlations between esds in cell parameters are only used when they are defined by crystal symmetry. An approximate (isotropic) treatment of cell esds is used for estimating esds involving l.s. planes.
Refinement. Refinement of F^2^ against ALL reflections. The weighted R-factor wR and goodness of fit S are based on F^2^, conventional R-factors R are based on F, with F set to zero for negative F^2^. The threshold expression of F^2^ > 2sigma(F^2^) is used only for calculating R-factors(gt) etc. and is not relevant to the choice of reflections for refinement. R-factors based on F^2^ are statistically about twice as large as those based on F, and R- factors based on ALL data will be even larger.

### Fractional atomic coordinates and isotropic or equivalent isotropic displacement parameters (Å^2^)

**Table:**

	*x*	*y*	*z*	*U*_iso_*/*U*_eq_	
Cl1	−0.00506 (8)	0.00115 (14)	0.29747 (5)	0.1569 (5)	
Cl2	0.10909 (6)	−0.10965 (8)	0.04695 (4)	0.1029 (3)	
O1	0.37565 (15)	−0.14366 (14)	0.07044 (8)	0.0792 (5)	
O2	0.22210 (16)	0.57128 (19)	0.35788 (8)	0.0895 (5)	
N1	0.60809 (12)	0.46755 (15)	0.06399 (7)	0.0491 (3)	
N2	0.55921 (13)	0.53315 (15)	0.11542 (7)	0.0503 (3)	
C1	0.29601 (17)	0.0166 (2)	0.21442 (10)	0.0587 (5)	
H1A	0.3728	0.0399	0.2281	0.070*	
C2	0.2134 (2)	0.0251 (3)	0.26089 (11)	0.0758 (6)	
H2A	0.2339	0.0536	0.3053	0.091*	
C3	0.1008 (2)	−0.0092 (3)	0.24031 (14)	0.0864 (8)	
C4	0.06896 (18)	−0.0522 (3)	0.17537 (14)	0.0815 (7)	
H4A	−0.0080	−0.0758	0.1623	0.098*	
C5	0.15265 (18)	−0.0601 (2)	0.12960 (11)	0.0630 (5)	
C6	0.26828 (15)	−0.02566 (18)	0.14784 (9)	0.0503 (4)	
C7	0.36214 (17)	−0.03724 (19)	0.09968 (10)	0.0544 (4)	
C8	0.43666 (16)	0.07857 (18)	0.08997 (10)	0.0556 (4)	
H8A	0.5025	0.0649	0.0666	0.067*	
C9	0.41725 (15)	0.20262 (18)	0.11213 (9)	0.0506 (4)	
H9A	0.3518	0.2145	0.1361	0.061*	
C10	0.48768 (14)	0.32072 (17)	0.10265 (9)	0.0484 (4)	
C11	0.56707 (15)	0.34103 (18)	0.05548 (9)	0.0521 (4)	
H11A	0.5886	0.2788	0.0235	0.063*	
C12	0.48548 (14)	0.44448 (17)	0.13854 (9)	0.0464 (4)	
C13	0.41627 (15)	0.47995 (17)	0.19521 (9)	0.0474 (4)	
C14	0.40070 (18)	0.3886 (2)	0.24752 (10)	0.0600 (5)	
H14A	0.4345	0.3035	0.2465	0.072*	
C15	0.3361 (2)	0.4226 (2)	0.30053 (10)	0.0692 (6)	
H15A	0.3265	0.3604	0.3350	0.083*	
C16	0.28559 (18)	0.5482 (2)	0.30297 (10)	0.0621 (5)	
C17	0.30101 (18)	0.6408 (2)	0.25277 (10)	0.0602 (5)	
H17A	0.2681	0.7263	0.2546	0.072*	
C18	0.36598 (16)	0.60618 (18)	0.19929 (10)	0.0536 (4)	
H18A	0.3760	0.6692	0.1653	0.064*	
C20	0.69679 (15)	0.5328 (2)	0.03000 (9)	0.0523 (4)	
C21	0.7582 (2)	0.4611 (2)	−0.01461 (12)	0.0750 (6)	
H21A	0.7418	0.3706	−0.0231	0.090*	
C22	0.8442 (2)	0.5251 (3)	−0.04652 (15)	0.0998 (9)	
H22A	0.8861	0.4773	−0.0769	0.120*	
C23	0.8693 (2)	0.6584 (3)	−0.03433 (15)	0.0954 (9)	
H23A	0.9278	0.7005	−0.0563	0.114*	
C24	0.8083 (2)	0.7289 (3)	0.01009 (13)	0.0868 (8)	
H24A	0.8260	0.8190	0.0191	0.104*	
C25	0.7199 (2)	0.6667 (2)	0.04183 (11)	0.0701 (6)	
H25A	0.6765	0.7155	0.0710	0.084*	
C19	0.1650 (3)	0.6978 (3)	0.36102 (15)	0.1027 (9)	
H19A	0.1246	0.7019	0.4016	0.154*	
H19B	0.1101	0.7081	0.3218	0.154*	
H19C	0.2217	0.7688	0.3618	0.154*	

### Atomic displacement parameters (Å^2^)

**Table:**

	*U*^11^	*U*^22^	*U*^33^	*U*^12^	*U*^13^	*U*^23^
Cl1	0.0996 (6)	0.2564 (14)	0.1240 (7)	0.0561 (7)	0.0633 (5)	0.0367 (8)
Cl2	0.0988 (5)	0.1161 (6)	0.0880 (5)	−0.0188 (4)	−0.0244 (4)	−0.0211 (4)
O1	0.1073 (12)	0.0476 (8)	0.0883 (11)	−0.0132 (8)	0.0403 (9)	−0.0177 (7)
O2	0.1016 (12)	0.1057 (14)	0.0668 (10)	−0.0030 (10)	0.0394 (9)	−0.0014 (9)
N1	0.0534 (8)	0.0454 (8)	0.0492 (8)	−0.0048 (6)	0.0081 (6)	−0.0045 (6)
N2	0.0573 (8)	0.0455 (8)	0.0490 (8)	−0.0038 (7)	0.0088 (6)	−0.0049 (6)
C1	0.0565 (10)	0.0626 (12)	0.0565 (11)	0.0029 (9)	0.0020 (8)	−0.0010 (9)
C2	0.0789 (14)	0.0938 (17)	0.0554 (12)	0.0208 (13)	0.0093 (10)	−0.0001 (11)
C3	0.0659 (13)	0.118 (2)	0.0787 (17)	0.0219 (13)	0.0242 (12)	0.0180 (15)
C4	0.0486 (11)	0.0994 (19)	0.0966 (19)	0.0007 (11)	0.0074 (11)	0.0119 (15)
C5	0.0606 (11)	0.0604 (12)	0.0665 (12)	−0.0041 (9)	−0.0029 (9)	0.0034 (9)
C6	0.0542 (9)	0.0415 (9)	0.0554 (10)	−0.0023 (7)	0.0057 (8)	0.0001 (7)
C7	0.0650 (11)	0.0438 (10)	0.0555 (10)	−0.0032 (8)	0.0108 (8)	−0.0046 (8)
C8	0.0578 (10)	0.0458 (10)	0.0648 (11)	−0.0031 (8)	0.0153 (8)	−0.0049 (8)
C9	0.0497 (9)	0.0469 (10)	0.0556 (10)	−0.0028 (7)	0.0071 (7)	−0.0034 (8)
C10	0.0489 (9)	0.0416 (9)	0.0548 (10)	−0.0016 (7)	0.0054 (7)	−0.0034 (7)
C11	0.0551 (9)	0.0448 (10)	0.0570 (11)	−0.0029 (8)	0.0079 (8)	−0.0092 (8)
C12	0.0498 (9)	0.0409 (9)	0.0482 (9)	−0.0011 (7)	0.0032 (7)	−0.0005 (7)
C13	0.0517 (9)	0.0438 (9)	0.0467 (9)	−0.0055 (7)	0.0050 (7)	−0.0024 (7)
C14	0.0737 (12)	0.0477 (11)	0.0587 (11)	0.0003 (9)	0.0058 (9)	0.0060 (8)
C15	0.0894 (15)	0.0674 (14)	0.0521 (11)	−0.0092 (11)	0.0145 (10)	0.0130 (9)
C16	0.0651 (11)	0.0712 (14)	0.0519 (11)	−0.0090 (10)	0.0161 (9)	−0.0051 (9)
C17	0.0676 (11)	0.0531 (11)	0.0622 (12)	0.0009 (9)	0.0184 (9)	−0.0050 (9)
C18	0.0636 (10)	0.0436 (10)	0.0551 (10)	−0.0034 (8)	0.0132 (8)	0.0018 (8)
C20	0.0517 (9)	0.0572 (11)	0.0478 (10)	−0.0065 (8)	0.0037 (7)	0.0017 (8)
C21	0.0760 (14)	0.0707 (14)	0.0826 (15)	−0.0097 (11)	0.0314 (12)	−0.0063 (12)
C22	0.0927 (18)	0.112 (2)	0.102 (2)	−0.0194 (17)	0.0487 (16)	−0.0093 (17)
C23	0.0801 (16)	0.119 (2)	0.0897 (19)	−0.0360 (16)	0.0247 (14)	0.0108 (16)
C24	0.0942 (17)	0.0819 (17)	0.0840 (17)	−0.0398 (14)	0.0068 (14)	0.0048 (13)
C25	0.0831 (14)	0.0645 (14)	0.0639 (13)	−0.0221 (11)	0.0124 (10)	−0.0057 (10)
C19	0.110 (2)	0.113 (2)	0.0925 (19)	0.0034 (18)	0.0490 (16)	−0.0307 (16)

### Geometric parameters (Å, °)

**Table:**

Cl1—C3	1.732 (2)	C11—H11A	0.9300
Cl2—C5	1.732 (2)	C12—C13	1.469 (2)
O1—C7	1.219 (2)	C13—C18	1.385 (2)
O2—C16	1.375 (2)	C13—C14	1.395 (3)
O2—C19	1.420 (3)	C14—C15	1.374 (3)
N1—C11	1.346 (2)	C14—H14A	0.9300
N1—N2	1.3652 (19)	C15—C16	1.378 (3)
N1—C20	1.423 (2)	C15—H15A	0.9300
N2—C12	1.329 (2)	C16—C17	1.372 (3)
C1—C2	1.377 (3)	C17—C18	1.386 (3)
C1—C6	1.387 (3)	C17—H17A	0.9300
C1—H1A	0.9300	C18—H18A	0.9300
C2—C3	1.367 (4)	C20—C25	1.371 (3)
C2—H2A	0.9300	C20—C21	1.372 (3)
C3—C4	1.367 (4)	C21—C22	1.373 (3)
C4—C5	1.377 (3)	C21—H21A	0.9300
C4—H4A	0.9300	C22—C23	1.370 (4)
C5—C6	1.390 (3)	C22—H22A	0.9300
C6—C7	1.502 (3)	C23—C24	1.361 (4)
C7—C8	1.456 (3)	C23—H23A	0.9300
C8—C9	1.331 (2)	C24—C25	1.384 (3)
C8—H8A	0.9300	C24—H24A	0.9300
C9—C10	1.446 (2)	C25—H25A	0.9300
C9—H9A	0.9300	C19—H19A	0.9600
C10—C11	1.373 (2)	C19—H19B	0.9600
C10—C12	1.418 (2)	C19—H19C	0.9600
			
C16—O2—C19	117.41 (19)	C18—C13—C12	121.17 (15)
C11—N1—N2	111.97 (14)	C14—C13—C12	121.21 (16)
C11—N1—C20	128.54 (15)	C15—C14—C13	120.91 (19)
N2—N1—C20	119.37 (14)	C15—C14—H14A	119.5
C12—N2—N1	104.76 (14)	C13—C14—H14A	119.5
C2—C1—C6	122.08 (19)	C14—C15—C16	120.39 (18)
C2—C1—H1A	119.0	C14—C15—H15A	119.8
C6—C1—H1A	119.0	C16—C15—H15A	119.8
C3—C2—C1	118.6 (2)	C17—C16—O2	124.4 (2)
C3—C2—H2A	120.7	C17—C16—C15	119.95 (18)
C1—C2—H2A	120.7	O2—C16—C15	115.61 (19)
C2—C3—C4	121.7 (2)	C16—C17—C18	119.53 (19)
C2—C3—Cl1	119.7 (2)	C16—C17—H17A	120.2
C4—C3—Cl1	118.7 (2)	C18—C17—H17A	120.2
C3—C4—C5	118.9 (2)	C13—C18—C17	121.60 (17)
C3—C4—H4A	120.5	C13—C18—H18A	119.2
C5—C4—H4A	120.5	C17—C18—H18A	119.2
C4—C5—C6	121.7 (2)	C25—C20—C21	120.49 (19)
C4—C5—Cl2	117.99 (17)	C25—C20—N1	119.80 (17)
C6—C5—Cl2	120.32 (16)	C21—C20—N1	119.71 (18)
C1—C6—C5	117.05 (17)	C20—C21—C22	119.0 (2)
C1—C6—C7	120.06 (16)	C20—C21—H21A	120.5
C5—C6—C7	122.85 (17)	C22—C21—H21A	120.5
O1—C7—C8	121.38 (17)	C23—C22—C21	121.0 (3)
O1—C7—C6	119.50 (17)	C23—C22—H22A	119.5
C8—C7—C6	119.10 (16)	C21—C22—H22A	119.5
C9—C8—C7	124.68 (17)	C24—C23—C22	119.7 (2)
C9—C8—H8A	117.7	C24—C23—H23A	120.1
C7—C8—H8A	117.7	C22—C23—H23A	120.1
C8—C9—C10	126.51 (16)	C23—C24—C25	120.1 (2)
C8—C9—H9A	116.7	C23—C24—H24A	120.0
C10—C9—H9A	116.7	C25—C24—H24A	120.0
C11—C10—C12	104.58 (15)	C20—C25—C24	119.6 (2)
C11—C10—C9	128.28 (16)	C20—C25—H25A	120.2
C12—C10—C9	127.08 (15)	C24—C25—H25A	120.2
N1—C11—C10	107.42 (15)	O2—C19—H19A	109.5
N1—C11—H11A	126.3	O2—C19—H19B	109.5
C10—C11—H11A	126.3	H19A—C19—H19B	109.5
N2—C12—C10	111.27 (15)	O2—C19—H19C	109.5
N2—C12—C13	120.38 (15)	H19A—C19—H19C	109.5
C10—C12—C13	128.34 (15)	H19B—C19—H19C	109.5
C18—C13—C14	117.61 (16)		
			
C11—N1—N2—C12	−0.55 (19)	C9—C10—C12—N2	−178.05 (16)
C20—N1—N2—C12	−176.84 (15)	C11—C10—C12—C13	−179.56 (17)
C6—C1—C2—C3	−0.1 (4)	C9—C10—C12—C13	3.1 (3)
C1—C2—C3—C4	0.4 (4)	N2—C12—C13—C18	40.9 (2)
C1—C2—C3—Cl1	−179.74 (18)	C10—C12—C13—C18	−140.33 (19)
C2—C3—C4—C5	−0.5 (4)	N2—C12—C13—C14	−137.87 (19)
Cl1—C3—C4—C5	179.7 (2)	C10—C12—C13—C14	40.9 (3)
C3—C4—C5—C6	0.1 (4)	C18—C13—C14—C15	1.0 (3)
C3—C4—C5—Cl2	−178.0 (2)	C12—C13—C14—C15	179.78 (18)
C2—C1—C6—C5	−0.3 (3)	C13—C14—C15—C16	−0.1 (3)
C2—C1—C6—C7	−178.16 (19)	C19—O2—C16—C17	2.7 (3)
C4—C5—C6—C1	0.2 (3)	C19—O2—C16—C15	−177.7 (2)
Cl2—C5—C6—C1	178.28 (15)	C14—C15—C16—C17	−0.9 (3)
C4—C5—C6—C7	178.1 (2)	C14—C15—C16—O2	179.47 (19)
Cl2—C5—C6—C7	−3.9 (3)	O2—C16—C17—C18	−179.4 (2)
C1—C6—C7—O1	125.1 (2)	C15—C16—C17—C18	1.0 (3)
C5—C6—C7—O1	−52.7 (3)	C14—C13—C18—C17	−0.9 (3)
C1—C6—C7—C8	−53.7 (3)	C12—C13—C18—C17	−179.66 (17)
C5—C6—C7—C8	128.5 (2)	C16—C17—C18—C13	−0.1 (3)
O1—C7—C8—C9	169.9 (2)	C11—N1—C20—C25	174.64 (19)
C6—C7—C8—C9	−11.3 (3)	N2—N1—C20—C25	−9.8 (3)
C7—C8—C9—C10	−178.89 (18)	C11—N1—C20—C21	−5.1 (3)
C8—C9—C10—C11	19.4 (3)	N2—N1—C20—C21	170.51 (18)
C8—C9—C10—C12	−163.87 (19)	C25—C20—C21—C22	0.9 (4)
N2—N1—C11—C10	0.1 (2)	N1—C20—C21—C22	−179.4 (2)
C20—N1—C11—C10	175.99 (17)	C20—C21—C22—C23	0.1 (4)
C12—C10—C11—N1	0.32 (19)	C21—C22—C23—C24	0.0 (5)
C9—C10—C11—N1	177.64 (17)	C22—C23—C24—C25	−1.1 (4)
N1—N2—C12—C10	0.75 (19)	C21—C20—C25—C24	−2.0 (3)
N1—N2—C12—C13	179.72 (15)	N1—C20—C25—C24	178.3 (2)
C11—C10—C12—N2	−0.7 (2)	C23—C24—C25—C20	2.1 (4)

### Hydrogen-bond geometry (Å, °)

**Table:**

*D*—H···*A*	*D*—H	H···*A*	*D*···*A*	*D*—H···*A*
C11—H11A···O1^i^	0.93	2.35	3.271 (2)	171

Symmetry codes: (i) −*x*+1, −*y*, −*z*.
